# A systematic review of co-responder models of police mental health ‘street’ triage

**DOI:** 10.1186/s12888-018-1836-2

**Published:** 2018-08-15

**Authors:** Stephen Puntis, Devon Perfect, Abirami Kirubarajan, Sorcha Bolton, Fay Davies, Aimee Hayes, Eli Harriss, Andrew Molodynski

**Affiliations:** 1 Department of Psychiatry, University of Oxford, Warneford Hospital, Oxford, OX3 7JX UK; 2 Oxford Health NHS Foundation Trust, Warneford Hospital, Oxford, OX3 7JX UK; 3 MD Program, University of Toronto Faculty of Medicine, Toronto, ON M5S 1A8 Canada; 4 Coventry and Warwickshire Partnership NHS Trust, Wayside House, Wilsons Lane Coventry, Warwickshire, CV6 6NY UK; 5 Bodleian Health Care Libraries, University of Oxford, Oxford, UK

**Keywords:** Police and mental health, Street triage, Mental health crisis, Crisis team

## Abstract

**Background:**

Police mental health street triage is an increasingly common intervention when dealing with police incidents in which there is a suspected mental health component. We conducted a systematic review of street triage interventions with three aims. First, to identify papers reporting on models of co-response police mental health street triage. Second, to identify the characteristics of service users who come in to contact with these triage services. Third, to evaluate the effectiveness of co-response triage services.

**Methods:**

We conducted a systematic review. We searched the following databases: Ovid MEDLINE, Embase, PsycINFO, EBSCO CINAHL, Scopus, Thompson Reuters Web of Science Core Collection, The Cochrane Library, ProQuest National Criminal Justice Reference Service Abstracts, ProQuest Dissertations & Theses, EThoS, and OpenGrey. We searched reference and citation lists. We also searched for other grey literature through Google, screening the first 100 PDFs of each of our search terms. We performed a narrative synthesis of our results.

**Results:**

Our search identified 11,553 studies. After screening, 26 were eligible. Over two-thirds (69%) had been published within the last 3 years. We did not identify any randomised control trials. Results indicated that street triage might reduce the number of people taken to a place of safety under S136 of the Mental Health Act where that power exists, or reduce the use of police custody in other jurisdictions.

**Conclusions:**

There remains a lack of evidence to evaluate the effectiveness of street triage and the characteristics, experience, and outcomes of service users. There is also wide variation in the implementation of the co-response model, with differences in hours of operation, staffing, and incident response.

**Electronic supplementary material:**

The online version of this article (10.1186/s12888-018-1836-2) contains supplementary material, which is available to authorized users.

## Background

Police officers routinely encounter people who are experiencing mental health crises. In the United Kingdom (UK), estimates of the proportion of police incidents linked to mental health crises range from as little as 2% to nearly 50% [1, 2]. This has been increasing over recent years both in the UK and internationally [2–6], yet police often feel that they lack the skills to appropriately support those in crisis [6, 7].

One approach to supporting police when they attend to people with mental health problems is through on-scene mental health triage. The general aim of these triage models is to introduce mental health expertise during the encounter in order to reduce the likelihood of the person in crisis being detained in police custody, and to reduce the distress caused to persons during these incidents [8]. An important parallel aim is to improve access to mental health treatment afterwards [9]. There are two main overarching models of triage: Police officers who have special mental health training (often referred to as Crisis Intervention Teams – CIT), or a co-response model where mental health professionals assist the police during incidents either in person or remotely from a control room.

The co-response model is the predominant model of police mental health triage in the UK. Despite its popularity with police and healthcare workers, it has been implemented without any meaningful investigation into its effectiveness. A previous review of policing mental health interventions identified only five published studies of street triage [10]. Their review, however, included only experimental or pre-post study designs and studies of effectiveness, excluding qualitative literature, epidemiological descriptions of the population, cost-effectiveness studies, and grey literature. Given the recent emergence of the street triage intervention, these other sources are likely to be informative. It also contrasted co-response models with other mental health police interventions, and did not describe the differences between co-response models. In this article, we systematically reviewed co-response models of police mental health street triage with three aims:

1. To identify and describe different co-response models of police mental health street triage.
2. To identify demographic and clinical characteristics of service users.
3. To evaluate the evidence for the effectiveness of co-response police mental health triage.

## Methods

### Criteria for selecting studies

All studies had to meet three criteria detailed in Table 1.

**Table 1:** Systematic review inclusion and exclusion criteria

Criteria	Sub criteria	
	Inclusion	Exclusion
1. A co-response model of police mental health triage	• Include both police officers and mental health workers in the response. • Be a response to a police incident.	
2. Describe a triage model or model development OR implementation of a triage model OR epidemiological study OR evaluation of effectiveness.	• Any original published article • Grey literature, service evaluations	• Review articles, book chapters, editorials or comments
3. English language article		

### Search strategy, study screening and selection

On the 30th April 2018, we carried out searches on the following databases: Ovid MEDLINE, Embase, PsycINFO, EBSCO CINAHL, Scopus, Thompson Reuters Web of Science Core Collection, The Cochrane Library, and ProQuest National Criminal Justice Reference Service Abstracts. We also searched grey literature via ProQuest Dissertations & Theses, EThoS, and OpenGrey. Finally, to obtain other grey literature we entered our search terms in to Google, limiting our search to the first 100 PDF results for each search term. A search strategy was developed for MEDLINE (see Additional file 1) and adapted for the other databases. The following free text terms and phrases were used to search the title and abstract fields to retrieve relevant literature: street triag*, policing or police and triag*, “mental health” and triag*, “liaison and diversion”, “speciali#ed mental health response*”, “co-responder”, coresponder*, “crisis intervention team*”, policing or police and “crisis team*”, “mental health” and “crisis team*”, policing or police and “mental health”, policing or police and liaison, “section 136”, or “psychiatric emergency response team*”.

As there is no consistent terminology for police mental health triage, these search strategies did not include MeSH terms. We mitigated this limitation by performing a second round of searches with a basic MeSH query to combine these sets of terms: (exp Mental Disorders/ or Triage/) and exp. POLICE/; Triage/ and exp. Mental Disorders/; Emergency Services, Psychiatric/ and exp. POLICE/. We inspected reference lists and forward citations of all included studies for further relevant literature.

Two teams of reviewers independently screened titles and abstracts (DP, AK, FD, AH). Any disagreements on eligibility were resolved through discussion with a third reviewer (SP). Following the title and abstract review, two reviewers conducted a full text review of eligible articles (DP, AK). We did not quality assess articles due to the broad range of methodologies included.

### Data analysis

We conducted pilot searches in preparation for this systematic review, and during these it became clear that there were likely to be few, if any, randomised controlled trials of street triage. Therefore, we decided to conduct a narrative synthesis of our results. We extracted data from eligible studies using a standardised data extraction form. We contacted authors to request necessary information on triage model design or results data if it was missing from the article. This included data on: authors and publication type, study design, sample size and demographics, type of intervention, days and hours of operation, details of how the service was operated and staff roles, and details of outcomes recorded. We then grouped studies with similar measures and outcomes together in themes. We divided eligible articles into research studies and non-academic reports and report on them separately.

## Results

**Fig. 1:**
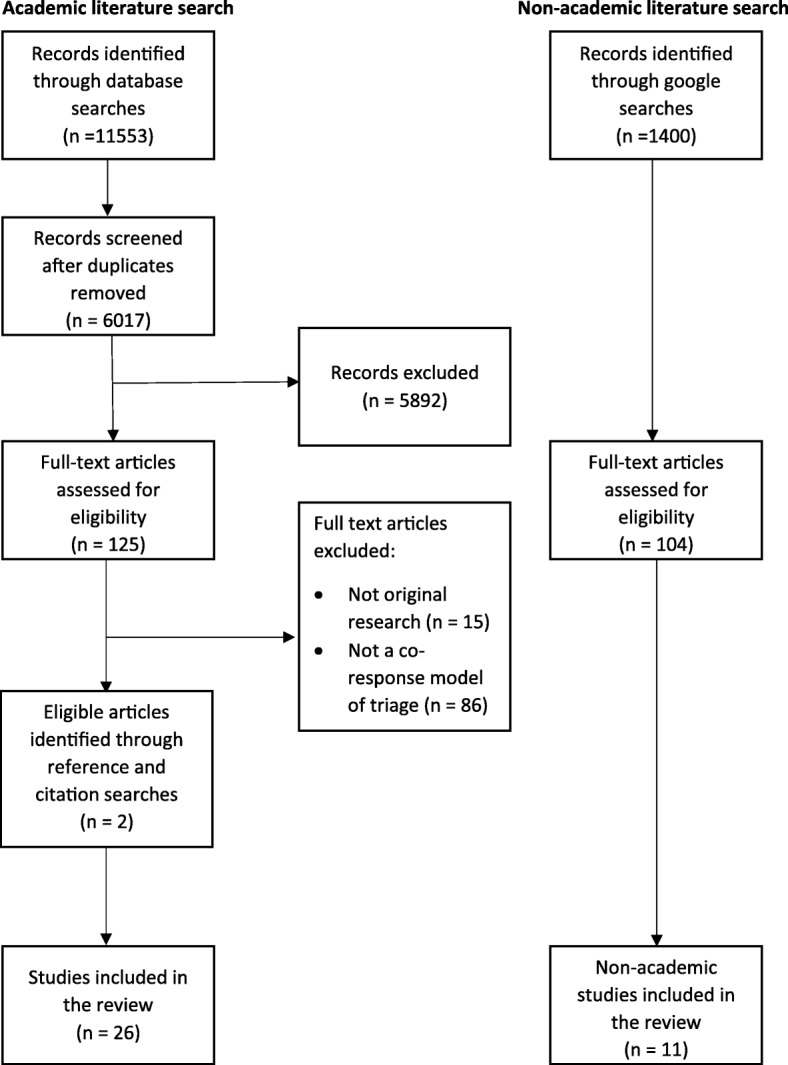
Flowchart of included studies

Our search produced 11,553 results. After removing duplicates, we screened the titles and abstracts of 6017 articles for relevance and considered 125 eligible for full text review (Fig. 1) (see Additional file 2). Twenty-four articles met full-text eligibility criteria, and we identified two further articles from searching the citations and reference lists of the eligible articles resulting in 26 articles of 23 studies included overall (Table 2) [11–36]. Our internet search for other grey literature produced 1400 results. Initial screening for relevance reduced this to 104 results, and a full-text review reduced this to 11 results.

**Table 2:** Description of co-response police mental health triage model and characteristics of included articles

Author	Year	Country	Study Design	Sample size	Follow-up (months)	Model Type	Times of Operation	Days of operation	Vehicle	Type of response
Boscarato et al.	2014	Australia	Qualitative	11	None	Not reported	Not reported	Not reported	Not reported	Not reported
Huppert & Griffiths	2015	Australia	Implementation	235	None	Ride-along model	15.00–23.00	7 days a week	Marked police car	Second response
Lee et al. and Evangelista et al.^1^	2015 & 2016	Australia	Mixed-methods	Quantitative: 296 Survey: 77 Qualitative: 12	6	Ride-along model	14.00–22.00	7 days a week^7^	Marked police car	Second response
Mckenna et al.	2015	Australia	Qualitative	17	None	Ride-along model	15.00–23.00	7 days a week	Marked police car^7^	Second response
Furness et al.	2017	Australia	Cross-sectional semi structured interview	43	None	Ride-along model	15.00–23.00	7 days a week	Marked police car^7^	Second response
Kisely et al.	2010	Canada	Mixed-methods	Quantitative: 2828 Qualitative: 84	24	Telephone support with non-uniformed police and clinician call-out if necessary	00.00–00.00	7 days a week	Ambulance & unmarked police car^7^	Second response
Kirst et al.	2015	Canada	Qualitative	54	None	Ride-along model	12 h^7^	7 days a week^7^	Marked police car	Second response
Fahim et al.	2016	Canada	Mixed-methods	Not reported	None	Ride-along model	Not reported	Not reported	Not reported	First response
Lamana et al.	2018	Canada	Mixed methods	See footnote^4^	None	Ride along model	12 h^7^	7 days a week^7^	Marked police car	Second response
Dyer et al.	2015	UK	Mixed-methods	Quantitative: 572 Qualitative: 16	None	Co-response nurses based at police station. Phone response. Secondary ride-along response	12.00–00.00	7 days a week	Unmarked car^7^	Both^7^
Heslin et al. & Heslin et al. ^2^	2015 & 2016	UK	Before and after & Health economics	55	12	Ride-along model	16.30–00.00 & 09.00–00.00	Wed - Fri & Sat - Sun	Unmarked police car	Both
Horspool et al.	2016	UK	Qualitative	15	None	1) Ride-along model. 2) Ride-along with telephone support	Varied	Varied	Not reported	Not reported
Jenkins	2016	UK	Before and after	N/a^5^	6	1) Ride-along model 2) Telephone support	1.) 14.00–00.00 2). 8.00–22.00	7 days a week	Marked car^7^	Both^7^
Keown et al.	2016	UK	Before and after	n/a^6^	None	Ride-along model	10.00–03.00	7 days a week	Unmarked car^7^	Both^7^
Lamb et al.	1995	USA	Retrospective case note review	101	6	Ride-along model	16 h a day (does not specify times)	7 days a week	Marked police car	Second response
Ligon & Thyrer	2000	USA	Cross sectional survey	83	None	Ride-along model	15.00–22.30	7 days a week	Marked police car	Both
Deane et al.	1999	USA	Survey	174	None	N/a Survey of MH provision	N/a Survey of MH provision	N/a Survey of MH provision	N/a Survey of MH provision	N/a Survey of MH provision
Scott	2000	USA	Mixed-methods	Quantitative: 131 Survey: 32	3	Ride-along model	15.00–22.30	7 days a week	Not reported	Both
Hails & Borum	2003	USA	Survey	135	None	N/a Survey of MH provision	N/a Survey of MH provision	N/a Survey of MH provision	N/a Survey of MH provision	N/a Survey of MH provision
Abbott	2011	USA	Survey	414	None	Ride-along model	16.00–00.00	Mon – Fri^7^	Marked police car	Both^7^
Iacoboni & Scott-Hayward	2015	USA	Process evaluation	33	None	Office-based with ride-along support	Not reported	Not reported	Marked police car	Second response
Lopez	2016	USA	Retrospective case note review	15,454	None	Office-based with ride-along support if necessary	Not reported	Not reported	Not reported	Not reported
Compton et al. & Compton et al.^3^	2017	USA	Qualitative & Feasibility study	Qualitative: 49 Feasibility: 199	0	Telephone support model	24 h a day	7 days a week	No car – police officer only	Second response

^1^These two articles are part of the same study

^2^These two articles are part of the same study

^3^These two articles are part of the same study

^4^This study used multiple datasets. It used two administrative datasets, the first *n* = 4607 investigated triage user characteristics, whilst the second compare outcomes from street triage incidents against police only incidence n = 18,969. The qualitative dataset *n* = 15

^5^This study investigated service-level outcomes and did not report number of street triage interventions

^6^This study investigated service-level outcomes and did not report number of street triage interventions, but did report rate of ST per 100,000 (138.7 per 100 k)

^7^We obtained this information from contacting study authors

All included articles had been conducted in Australia, Canada, the United States of America (USA) or the United Kingdom, and more than two-thirds (*n* = 18, 69%) had been published from 2015 onwards. We did not identify any randomised controlled trials. Six articles used mixed-methods, six had qualitative methodology, four surveys, three before-and-after studies, two retrospective case-note reviews, one cross-sectional semi-structured interview, one process evaluation, one implementation study, one feasibility study, and one health economics paper. Six articles (23%) measured data at more than one time point, with follow-up durations between 3 and 24 months.

### Service models

There were 19 different triage models described in the 26 articles (Table 2). Twelve used a ride-along model, where police officer and mental health worker attend the incident in the same vehicle. Five were services with both ride-along and control room support, in which the mental health worker assists the officers remotely via telephone or police radio. Four of these described a service which used the control room as their main method of response, only using a mobile unit for particularly severe incidents. Two services used telephone triage as the method of response. We were unable to determine the method of one study due to a lack of adequate description, and two studies were national surveys which did not request details of the triage model.

We were able to identify how incidents were reported to the triage team for 15 models. Two took calls from emergency control rooms (e.g. 911, or a police control room), four took calls directly from police officers in the field, eight took calls from both emergency lines and police officers, and one had a direct line that emergency services, members of the public, and relevant agencies could call. Of the 15 models that reported details of the vehicles used in triage calls, nine used marked police cars, three an unmarked car, and one used an ambulance alongside an unmarked car (two models were telephone only models). Nine models described a system whereby the mobile unit was dispatched only when a normal police unit had already responded and determined the incident was safe, while one described the mobile unit acting as a first response to an incident and six used a combination of both methods of response. Data on type of response was missing from three models and the two remaining articles were the national surveys which did not collect data on the model of triage.

Twelve models had triage services that were operated 7 days a week, but only the telephone support models were run 24 h a day. Three models had less than 7 days a week services. Times of operation varied greatly (see Table 2), but most services were contracted to cover evening and night-time hours only.

### Service user characteristics

#### Demographics

Eight studies reported service users’ age [21, 23, 27, 29, 31, 32, 34, 35]. Four of the eight reported mean age; 39 [27], 37.3 [31], 35.7 [29], and 40.0 [32] years respectively. One study reported mean age in pre and post intervention cohorts between 34.7 and 37.7 years [23], one reported that 46% were between ages 18 and 39 [21], whilst two categorised age into ranges, where the highest percentage lay between the 35–44 (28%) and 35–54 (34.9%) age categories respectively [34, 35]. More males than females were referred to the triage services, with percentages between 47 and 77% reported in eight studies [21, 23, 27, 29, 31, 32, 34, 35]. Four studies, all from the USA, measured ethnicity [27, 29, 31, 32]. In two studies the most frequent reported service user ethnictiy was African-American, 65% [31], and 61.3% [32], with both studies taking place in the US state of Georgia, whilst in two studies, both undertaken in California, White ethnicity was the most frequent at 44.5% [27], and 33.8% [29].

#### Previous mental health service use

Three studies reported on the proportion of service users who had previously been in contact with mental health services. Over half of all referrals (51%) to the Cleveland Police Street Triage service were known to the local mental health team whilst 19% were currently on the caseload of a mental health service [14]. Similarly, 48% of those who received an intervention from the Police Ambulance Crisis Response (PACER) team in Melbourne [21], and 78% of service users seen by a Los Angeles triage team had a history of psychiatric hospitalisation [27]. Between 44 and 65% of people in Jenkins and colleagues’ study had had contact with a community mental health team within the past 2 weeks [23]. Diagnosis was recorded in three studies [32, 34, 35], with psychotic illnesses the most common reported diagnosis (between 26.0–42.9%) followed by mood disorders (between 9 and 32.5%).

### Co-response team involvement

#### Reason for triage response

The reason for triage response was only reported in three studies. The most common reason in two studies, responsible for 49% of all call-outs in Kisely and colleagues study [26], and 55% in Lee and colleagues’ [28], was concern for an individual’s welfare due to suicidal behaviour. In Lamb and colleagues’ study, the most common reason was “bizarre or disorganised behaviour” in 71% of call-outs [27].

#### Repeat use of triage

Four studies reported on repeat referrals to the service. Dyer and colleagues found that 12% of triage service users had a subsequent referral over an 18 month period [14], Huppert and colleagues found a rate of 13% over 3 months [21], Kisely and colleagues found a 20% rate over 36 months [26], whilst Lamana and colleagues found a 29.9% repeat referral rate over 12 months [35].

### Effectiveness of co-response police mental health triage

Many of the included studies did not look at effectiveness or outcome. Those that did took a varied approach and we report on a variety of aspects of effectiveness as a result.

#### Reduction in police detentions

Five studies measured changes in detention rates after the introduction of a co-response model. Four of these five studies compared the number of Section 136 s (S136, a police power in England and Wales which allow a police officer to detain someone whom they suspect to be mentally ill) either before and after the introduction of a co-response triage team [18, 23, 24] or in comparison to when the co-response team was not on duty [14]. All found that the co-response model decreased the number of service users made subject to S136. Jenkins and colleagues compared mental health workers paired with frontline police versus mental health support from a control room and found that the face-to-face model had a significant reduction in S136 after it was introduced whilst the control room model did not [23].

Two studies compared detention in custody during co-response operational times versus operational times without co-response [19, 29]. Heslin and colleagues found a significant reduction in mental health detentions in custody whilst Lopez found that of contacts coded in the police records as ‘mental health-related’, 1.4% of co-response contacts resulted in arrest whilst 13.3% of police-only contacts result in arrest.

#### Reduction in psychiatric hospitalisation

The introduction of a co-response team resulted in a reduction in the proportion of police incidents resulting in psychiatric hospitalisation in three studies [14, 18, 31], whilst one study found an overall reduction in hospitalisation due to fewer police detentions [24]. Three studies found an increase of psychiatric hospitalisation following the introduction of street triage [16, 23, 29]. One study compared a co-responding team to a usual response and found that whilst the co-response model was more likely to lead to escort to an emergency department, these were less likely to be involuntary escorts [35], whilst another study found that once participants were involuntarily hospitaised, participants reported better percieved procedural justice but no difference in percieved coercion from the admission event [34].

#### Perceptions of service users

Eight studies reported on the views of those who had experienced a co-response intervention [12, 14, 15, 25, 26, 30, 33, 35]. In five studies, participants reported that previous interactions with the police were traumatic [12, 14, 15, 30, 35] and in four how their illness had been treated as a criminal matter rather than a mental health one [12, 15, 30, 35]. In comparison, co-response models were better at de-escalation, less threatening, and less stigmatising [12, 14, 15, 25, 26, 30, 35]. Service users in one study suggested that the use of unmarked police cars and non-uniformed officers would further reduce distress and embarrassment [15].

Co-response services were reported as being more responsive to crises, with mental health workers on the scene immediately rather than a wait for police transport to a treatment destination or waiting for mental health personnel from a crisis team [12, 14, 33].

Three studies reported criticisms related to the lack of follow-up and case management. Service users often felt that there were not effective pathways to further treatment following the intervention [14, 15, 25].

#### Perceptions of providers

Nine studies reported on the service providers’ views of the co-responder models: three surveys, two qualitative studies, and four mixed-method designs [11, 13, 14, 16, 17, 25, 26, 28, 30]. Service providers found the service helpful and valued the service [11, 25, 26, 28], and reported improved coordination and collaboration between police, mental health services, and emergency departments [25, 28, 30]. They also reported improved speed and clarity of pathways to treatment for those seen by the triage teams [14, 16, 26, 28, 30]. The main criticism was the availability of the units, due to either limited operational hours or limited capacity [26, 28].

Three surveys reporting on police officers’ perception of different triage models found no difference between perceptions of police staff on effectiveness of the service, ability to respond to those in crisis, stigma, or level of mental health training [11, 13, 17].

#### Cost effectiveness

Three studies reported on the cost-effectiveness of street triage [18, 19, 31]. The average cost per crisis response was calculated to be 23% lower with the introduction of the triage programme in one analysis [31]. This was due to a reduction in hospitalisation. Heslin and colleagues found reduced overall costs due to reduced police costs, but with a smaller increase in health provider costs [19]. In a separate study by Heslin and colleagues, overall costs increased, but by less than 1% [18].

### Non-academic reports

Nine of the eligible documents were evaluations of a local street triage service [37–45], one was a report on a service for strategic review [41], and one was a report on a pilot scheme of nine services [42]. Eight were from the UK, two from the USA, and one from Australia. Reports differed in both methodology and methodological quality. Eight articles measured changes to S136 before and after the introduction of a service (seven local and one wider evaluation) [37–42, 46, 47]. Five local evaluations reported a decrease in the use of S136 [37, 38, 40, 41, 46] and two an increase [39, 42]. The report of nine evaluations found seven services with a decrease in S136 and two with an increase in S136 [47]. Five documents investigated detentions in police custody, with four finding a reduction in the use of custody [38, 39, 41, 42], and one finding no difference (although the use of custody was negligible both before and after introduction of the service) [38].

Nine documents reported on qualitative findings (eight local street triage services and the evaluation of nine sites described above) [37, 39–44, 47]. Four interviewed police, three interviewed mental health staff, two interviewed service users, and one interviewed carers. In the report of the nine pilot sites, each site conducted different interviews with different methodologies and different samples. Results were similar to those of the academic studies. Service users found the triage service less distressing than standard police responses [37, 39]. Police officers valued the service and found it reduced the time spent at incidents [37, 38, 42, 43, 47]. Areas identified for improvement included longer operational times [37, 43, 47], and more resources [43, 47].

## Discussion

The number of publications reporting on co-response models of police mental health triage has surged, with more than two-thirds of the studies included in this review published in the last 3 years. The co-response model of triage is now an established intervention in the US, Australia, and Canada, and has become the dominant model of mental health crisis response used by the police in the UK.

Overall, we found that co-response models were associated with a reduction in the use of police powers of detention (such as the use of S136) a reduction in detainment in police custody. Unfortunately, due to the design of the studies one cannot determine whether this is due to the intervention or other factors such as changes in policy, changes in mental health provision, or greater public scrutiny of mental health detentions [48]. Evidence from the qualitative studies we included in this review suggests that service users found the co-response interventions less distressing and less criminalising than a standard police response. Service users also reported quicker access to mental health support at the time of crisis, although this did not extend to gaining access to mental health services at follow-up.

In conducting this review, we identified three major limitations of the current evidence for co-response triage, a) the lack of information on the characteristics of service users b) the lack of detail when describing co-response models and the variation in their operationalisation and, c) the lack of rigorous comparative research on effectiveness.

### The lack of information on characteristics of service users

Few studies measured the demographic and clinical characteristics of triage service users or their reason for referral. From the data reported, those who are middle-aged and male are slightly overrepresented, as are those with psychotic illnesses and previous mental health history. The data we identified in both the academic and non-academic literature suggest that the majority of service users are already know to mental health teams, and between 10 and 20% have multiple referrals to the triage team.

Whilst psychiatric inpatient beds have declined over the last 60 years, the provision of forensic beds and the proportion of the mentally ill in prison has increased in both the US and Europe [5, 49]. Deinstitutionalisation without appropriate community treatment may have led to increased criminalisation of the mentally ill [50]. Police triage services are designed to divert those with mental illness away from police custody, but providing appropriate support prior to a crisis point may reduce the need for an intervention by emergency services [51]. There remains, however, a group of service users who come into contact with triage who have no previous mental health service use or known psychopathology. Measuring who is referred, their history leading up to referral, and the nature of the referral may improve our understanding of this vulnerable population. This may in turn allow us to be clear about the circumstances in which the use of the police-triage resource is appropriate and those in which it can be avoided. This could have significant implications for service use and most importantly for patient experience and outcome.

### Variations in the co-response model

Many of the papers included in this review did not adequately describe their model of triage. Less than half reported operational hours and only two-thirds described their method of response to incidents. When contacting the authors, many of these details were available, which suggests issues of how triage is reported in the literature rather than poor model specification.

There was marked variation in how co-response models were operationalised. There were differences in times and days of operation, whether the unit was a first or second-response option, whether the police officer and mental health worker were co-located, whether a mobile unit was dispatched or not, and the mode of transportation to the incident (marked or unmarked vehicles). There was also limited, if any, information on other mental health provision in the study area.

Local context will always inform model implementation, and rightly so. There must, however, be efforts to understand which components of the model are most effective and most acceptable to service users, and equally which are not. Where good practice is identified, such as the use of unmarked cars and non-uniformed police officers, these can be rigorously tested.

The current heterogeneity in model implementation creates a problem of circularity, whereby service providers cannot follow good practice guidelines because there are none. At the same time, commissioners cannot establish good practice because each model is idiosyncratic and cannot easily be compared.

This may in part be due to the lack of precise reporting of co-response models. Reporting aspects such as the location and composition of members of the team, hours of operation, referral processes, all potential response methods, and mode of transportation should all be reported as a minimum dataset. In Table 3, we suggest a framework for reporting triage models in future studies to enable model comparison.

**Table 3:** A suggested framework for collecting and reporting co-response triage models

Topic	Variable to report	Description of variable and reason for reporting
Identify as co-response triage service	Name of service	Give the name of the service to enable grouping of models and their comparison.
	Identify as co-response model of triage	Include a sentence in the service description to signpost to readers and researchers that the service is a co-response model of police mental health triage.
Model characteristics	Model of co-response	Define the model of how the mental health professional assists the police officer during the incident. *Ride-along* models are those in which the police officer (PO) and mental health (MH) worker attend the incident together in a vehicle. *Ride-separate* models are when PO and MH worker arrive at the incident separately. *Telephone Support* models are those in which the MH worker provides assistance via a telephone or radio. Services that provide a combination of these models should specify when each model is used.
	Method of referral to co-response team	Describe how crisis incidents are referred to the triage team (e.g. emergency response, direct from police officers, publicly available direct phone line), and from whom triage can take referrals (e.g. police officers, other emergency services, mental health services, the public, etc.)
	Timing of response	Describe whether the co-response team acts as a *first responder*, where the team can be referred to and attend incidents prior to any other police involvement, or *second responder*, where the team is referred following an initial police response.
	Team staffing and roles	Outline the team composition and responsibilities
	Days and hours of operation	Describe the working hours of members of the co-response team. Be explicit if hours differ for different types of response, or members of response.
	Team Location	Describe where each member of the team is located during a usual triage shift.
	Vehicle involved in triage response (if any)	If applicable, describe how the co-response team attend the triage incident (e.g. in a marked police car, unmarked car, ambulance, etc.)
Local context	Geography and population	Describe the geographic elements (e.g. rural versus city, large area vs small area) and local population to improve readers’ understanding of the local context in to which the triage service is placed.
	Local mental health provision and linked partner agencies	Describe local mental health provision and agencies working closely with the triage team (if any) to improve understanding of where triage fits within crisis services and wider mental health provision

### The lack of controlled research on the effectiveness of co-response models of police mental health triage

There is a striking lack of evidence for the effectiveness of co-response models of triage despite the substantial investment these services have received. We did not identify any randomised control trials. We identified one controlled before-and-after study, which found better engagement with mental health services following the intervention, but did not measure other important clinical follow-up data such as hospitalisation [26].

There would be significant challenges conducting a randomised trial of street triage, such as lack of blinding, consenting participants, and maintaining model fidelity. Whilst these difficulties are not insurmountable, other designs (such as longitudinal data with propensity score matching) may be more feasible. For instance, a quasi-experimental design such as propensity score matching could avoid the difficulty of gaining consent prior to randomisation by retrospectively matching those who have received a triage intervention with a sample who have not. This would incur less cost than an appropriate cluster randomised controlled trial, but would remain only an approximation of randomisation and would require careful covariate selection.

This lack of evidence needs to be urgently addressed as we currently have little understanding of both the immediate and the long-term clinical and functional outcomes following a triage intervention, nor any identification of potential adverse events resulting from it.

### Strengths and limitation

This is the first systematic review dedicated solely to investigating co-response street triage. We used a search strategy which included broad criteria and included many different databases and grey literature searches to ensure we captured all studies of street triage. We excluded reviews, book chapters, editorials, and non-English language articles which may have resulted in us missing some relevant literature.

We were unable to assess risk of bias due to the broad range of methodologies used in our included studies, and we decided to include this heterogeneous mix of studies rather than focus on any single methodology due to the sparse literature.

## Conclusion

Preliminary evidence suggests that co-response models of mental health police triage may reduce the use of police powers of detention on people with mental illness and be more acceptable to service users than a standard response. However, there remains a lack of quality of reporting in studies, few controlled studies investigating the effectiveness of co-response triage, and a lack of focus on the characteristics and outcomes of service users.

There are many unanswered questions about the effectiveness of police mental health triage. Is a police response the most appropriate response for people in mental health crisis? Alternatively, would an ambulance-based mental health crisis response be more effective, and more acceptable, than a police one? Is the perceived increased need for police mental health intervention due to a lack of appropriate mental health support elsewhere in the healthcare system? How can we better care for those who repeatedly make use of the triage service, for their benefit and for other users who need access?

Given the considerable recent investment of resources by police and mental health services, thoughtful evaluation of triage services should lead development of models rather than be left as an afterthought. Rigorous data on outcomes, both immediate and long-term, following a triage intervention is needed. We also need further exploration of service users and their carers’ experience of triage, and their participation in the design of these services. Finally, we need to move towards better model description and evaluation, with the aim of creating fidelity indicators linked to good practice and good outcomes.

## Additional files

Additional file 1: Search strategy. Systematic review search strategy in detail. (DOCX 21 kb)

Additional file 2: Papers excluded at full text review. Reference list of all the papers excluded during the full-text review stage. (DOCX 30 kb)

## Abbreviations

CIT: Crisis Intervention Team
MH: Mental health
PACER: Police Ambulance Crisis Response
PDF: Portable Document Format
PO: Police officer
UK: United Kingdom
