# Supplementary material for: A systematic review of co-responder models of police mental health ‘street’ triage
Source: BMC Psychiatry. 2018 Aug 15;18:256. doi: 10.1186/s12888-018-1836-2 (PMC6094921; doi:10.1186/s12888-018-1836-2)
Supplement: Supplementary file 1 — Search strategy. Systematic review search strategy in detail. (DOCX 21 kb) [file 12888_2018_1836_MOESM1_ESM.docx]

## Additional file 1: Search Strategy

## Mental Health Street Triage: Review

**SEARCH DATE: 21/03/17 – and re-run on 30/04/18**

**All searches designed and performed by Eli Harriss, Bodleian Health Care Libraries**

## Search Results

|  | **21/03/17** | **30/04/18** |
| --- | --- | --- |
| **Medline** | **1229** | **1290** |
| **Embase** | **1505** | **1693** |
| **Psycinfo** | **1662** | **1834** |
| **Cinahl** | **513** | **625** |
| **Scopus** | **1868** | **2080** |
| **Web of Science – Core Collection** | **1535** | **1834** |
| **The Cochrane Library** | **77** | **81** |
| **Proquest National Criminal Justice Reference Service (NCJRS) Abstracts** | **1604** | **1589** |
| **BL EThOS** | **54** | **57** |
| **OpenGREY** | **21** | **21** |
| **Proquest Theses and Dissertations Global** | **437** | **449** |
| **TOTAL** | **10477** | **11553** |
| **TOTAL AFTER DEDUPLICATION** | **5475** |  |
| **Unique since 21/03/17** | **-** | **623** |

## Search Strategies

### Ovid MEDLINE(R) Epub Ahead of Print, In-Process & Other Non-Indexed Citations, Ovid MEDLINE(R) Daily and Ovid MEDLINE(R) 1946 to Present

### Embase 1974 to 2018 April 27

### PsycINFO 1967 to April Week 4 2018

### EBSCO Cinahl

### Scopus

### The Cochrane Library: Cochrane Database of Systematic Reviews : Issue 4 of 12, April 2018

### Proquest National Criminal Justice Reference Service (NCJRS) Abstracts Database

### ProQuest and Dissertations and Theses (Global full text plus UK and Ireland abstracts)

((street and triag*) or ((police or policing) and triag*) or ("mental health" and triag*) or "liaison and diversion" or "speciali#ed mental health response*" or "co-responder*" or coresponder* or "crisis intervention team*" or ((police or policing) and CIT) or ("mental health" and CIT) or ((police or policing) and "crisis team*") or ("mental health" and "crisis team*") or ((police or policing) and "mental health") or ((police or policing) and liaison) or "section 136" or "psychiatric emergency response team*").ti,ab.

### Web of Science Core Collection

**TOPIC:** (((street and triag*) or ((police or policing) and triag*) or ("mental health" and triag*) or "liaison and diversion" or "speciali#ed mental health response*" or "co-responder*" or coresponder* or "crisis intervention team*" or ((police or policing) and CIT) or ("mental health" and CIT) or ((police or policing) and "crisis team*") or ("mental health" and "crisis team*") or ((police or policing) and "mental health") or ((police or policing) and liaison) or "section 136" or "psychiatric emergency response team*")) *OR* **TITLE:** (((street and triag*) or ((police or policing) and triag*) or ("mental health" and triag*) or "liaison and diversion" or "speciali#ed mental health response*" or "co-responder*" or coresponder* or "crisis intervention team*" or ((police or policing) and CIT) or ("mental health" and CIT) or ((police or policing) and "crisis team*") or ("mental health" and "crisis team*") or ((police or policing) and "mental health") or ((police or policing) and liaison) or "section 136" or "psychiatric emergency response team*"))

### EThOS (Electronic Theses Online Service)

Street triage

Police triage

Policing triage

Mental health triage

“liaison and diversion”

Specialised mental health response

Specialized mental health response

“crisis intervention team”

Police CIT

Policing CIT

Police crisis team

Policing crisis team

“mental health” “crisis team”

Police “mental health”

Policing “mental health”

Police liaison

Policing liaison

“section 136”

Psychiatric emergency response team

### OpenGREY

Street triage

Police triage

Policing triage

Mental triage

“liaison and diversion”

Specialised mental health response

Specialized mental health response

Co-responder

Coresponder

crisis intervention team

Police CIT

Policing CIT

Police crisis team

Policing crisis team

mental health crisis team

Police “mental health”

Policing “mental health”

Police liaison

Policing liaison

“section 136”

Psychiatric emergency response team
