# Supplementary material for: A systematic review of co-responder models of police mental health ‘street’ triage
Source: BMC Psychiatry. 2018 Aug 15;18:256. doi: 10.1186/s12888-018-1836-2 (PMC6094921; doi:10.1186/s12888-018-1836-2)
Supplement: Supplementary file 2 — Papers excluded at full text review. Reference list of all the papers excluded during the full-text review stage. (DOCX 30 kb) [file 12888_2018_1836_MOESM2_ESM.docx]

**Additional file 2: Papers excluded at full-text review**

Acker, J. A. T. (2010). *The effect of crisis intervention team training on the outcomes of mental health*

*crises calls for law enforcement* (Doctoral dissertation, Walden University).

Addy, C. (2005). An examination of the development of standardized training protocols for law

enforcement crisis intervention teams for the mental health consume, *Dissertation Abstracts*

*International Section A: Humanities and Social Science,* 65(11-A), 4111.

Barcelos, N. M. (2014). *The impact of Crisis Intervention Team (CIT) training on law enforcement*

*officers in connecticut*. University of Hartford, Connecticut.

Bard, M., & Berkowitz, B. (1969). A community psychology consultation program in police family crisis

intervention: Preliminary impressions. *International Journal of Social Psychiatry*, *15*(3), 209-

215.

Barillas, M. E. (2012). *Police officers as first line responders: Improving mental health training to*

*effectively serve the mentally ill population*. Alliant International University.

Beigel, A. (1973). Law enforcement, the judiciary, and mental health: A growing partnership.

*Psychiatric Services*, *24*(9), 605-609.

Blevins, K. R., Lord, V., & Bjerregaard, B. (2014). Evaluating Crisis Intervention Teams: possible

impediments and recommendations. *Policing: An International Journal of Police Strategies &*

*Management*, *37*(3), 484-500.

Bonfine, N., Ritter, C., & Munetz, M. R. (2014). Police officer perceptions of the impact of crisis

intervention team (CIT) programs. *International journal of law and psychiatry*, *37*(4), 341-350.

Borum, R. (2000). Improving high risk encounters between people with mental illness and the police.

*Journal of the American Academy of Psychiatry and the Law*, *28*(3), 332-337

Borum, R., Williams Deane, M., Steadman, H. J., & Morrissey, J. (1998). Police perspectives on

responding to mentally ill people in crisis: Perceptions of program effectiveness. *Behavioral*

*sciences & the law*, *16*(4), 393-405.

Bouveng, O., Bengtsson, F. A., & Carlborg, A. (2017). First-year follow-up of the Psychiatric

Emergency Response Team (PAM) in Stockholm County, Sweden: A descriptive study.

*International Journal of Mental Health*, *46*(2), 65-73.

Bower, D. L., & Pettit, W. (2001). The Albuquerque police department's crisis intervention team: A

report card. *FBI L. Enforcement Bull.*, *70*, 1.

Broussard, B., McGriff, J. A., Neubert, B. N. D., D’Orio, B., & Compton, M. T. (2010). Characteristics

of patients referred to psychiatric emergency services by crisis intervention team police

officers. *Community mental health journal*, *46*(6), 579-584.

Browning, S. L., Van Hasselt, V. B., Tucker, A. S., & Vecchi, G. M. (2011). Dealing with individuals

who have mental illness: The crisis intervention team (CIT) in law enforcement. *The British*

*Journal of Forensic Practice*, *13*(4), 235-243.

Camille-McKiness, K. (2013). *Police perspectives on CIT training: An ethnographic study of law*

*enforcement officers' perspectives on Crisis Intervention Team training* (Doctoral dissertation,

Northern Illinois University).

Campbell, R (2012) Evaluation of Chicago Police Department’s Crisis Intervention Team for Youth Training, https://bit.ly/2H2XbYz

Canada, K. E., Angell, B., & Watson, A. C. (2010). Crisis intervention teams in Chicago: successes on

the ground. *Journal of Police Crisis Negotiations*, *10*(1-2), 86-100.

Carrier, J. T. W. (1993). Evaluation of police crisis intervention in mental disturbance calls. (Doctoral dissertation, Memphis State University)

Compton, M. T., & Chien, V. H. (2008). Factors related to knowledge retention after crisis intervention

team training for police officers. *Psychiatric Services*, *59*(9), 1049-1051.

Compton, M. T., Bakeman, R., Broussard, B., Hankerson-Dyson, D., Husbands, L., Krishan, S., ... &

Watson, A. C. (2014). The police-based crisis intervention team (CIT) model: I. effects on

officers’ knowledge, attitudes, and skills. *Psychiatric services*, *65*(4), 517-522.

Compton, M. T., Bakeman, R., Broussard, B., Hankerson-Dyson, D., Husbands, L., Krishan, S., ... &

Watson, A. C. (2014). The police-based crisis intervention team (CIT) model: II. Effects on

level of force and resolution, referral, and arrest. *Psychiatric services*, *65*(4), 523-529.

Compton, M. T., Broussard, B., Hankerson-Dyson, D., Krishan, S., & Stewart-Hutto, T. (2011). Do

empathy and psychological mindedness affect police officers' decision to enter crisis

intervention team training?. *Psychiatric Services*, *62*(6), 632-638.

Compton, M. T., Broussard, B., Hankerson-Dyson, D., Krishan, S., Stewart, T., Oliva, J. R., & Watson,

C. (2010). System-and policy-level challenges to full implementation of the Crisis

Intervention Team (CIT) model. *Journal of Police Crisis Negotiations*, *10*(1-2), 72-85.

Compton, M. T., Broussard, B., Reed, T. A., Crisafio, A., & Watson, A. C. (2015). Surveys of police

chiefs and sheriffs and of police officers about CIT programs. *Psychiatric Services*, *66*(7),

760-763.

Compton, M. T., Demir, B., Oliva, J. R., & Boyce, T. (2009). Crisis intervention team training and

special weapons and tactics callouts in an urban police department. *Psychiatric Services*,

*60*(6), 831-833.

Compton, M. T., Esterberg, M. L., McGee, R., Kotwicki, R. J., & Oliva, J. R. (2006). Crisis intervention

team training: Changes in knowledge, attitudes, and stigma related to schizophrenia.

*Psychiatric Services*, *57*(8), 1199-1202.

Cowell, A. J., Hinde, J. M., Broner, N., & Aldridge, A. P. (2015). The cost of implementing a jail

diversion program for people with mental illness in San Antonio, Texas. *Evaluation and*

*program planning*, *48*, 57-62.

Cross, A. B., Mulvey, E. P., Schubert, C. A., Griffin, P. A., Filone, S., Winckworth-Prejsnar, K., ... &

Heilbrun, K. (2014). An agenda for advancing research on crisis intervention teams for mental

health emergencies. *Psychiatric Services*, *65*(4), 530-536.

Cuddeback, G. S., Kurtz, R. A., Blank Wilson, A., VanDeinse, T., & Burgin, S. E. (2016). Segmented

Versus Traditional Crisis Intervention Team Training. *J Am Acad Psychiatry Law*, *44*, 338-43.

Cummins, I., & Edmondson, D. (2016). Policing and street triage. *The Journal of Adult Protection*,

*18*(1), 40-52.

Earl, F., Cocksedge, K., Morgan, J., & Bolt, M. (2017). Evaluating liaison and diversion schemes: an

analysis of health, criminal and economic data. *The Journal of Forensic Psychiatry &*

*Psychology*, *28*(4), 562-580.

Ellis, H. A. (2011). The Crisis Intervention Team—A Revolutionary Tool for Law Enforcement: The

Psychiatric-Mental Health Nursing Perspective. *Journal of psychosocial nursing and mental*

*health services*, *49*(11), 37-43.

Ellis, H. A. (2014). Effects of a crisis intervention team (CIT) training program upon police officers

before and after crisis intervention team training. *Archives of psychiatric nursing*, *28*(1), 10-16.

El-Mallakh, P. L., Kiran, K., & El-Mallakh, R. S. (2014). Costs and savings associated with

implementation of a police crisis intervention team. *Southern Medical Journal*, *107*, 391.

El-Mallakh, R. S., Spratt, D., Butler, C., & Strauss, G. (2008). Evaluation of consequences of

implementation of police crisis intervention team in Louisville. *The Journal of the Kentucky*

*Medical Association*, *106*(9), 435-437.

El-Mallakh, R. S., Wulfman, G., Smock, W., & Blaser, E. (2003). Implementation of a crisis

intervention program for police response to mental health emergencies in Louisville. *The*

*Journal of the Kentucky Medical Association*, *101*(6), 241.

Erickson, B. R. (2018). *Discretionary Acts Fueled by Bureaucratic Anxieties: The Policing of*

*Community-Disrupting Mental Illness* (Doctoral dissertation, UCLA).

Erstling, S. S. (2006). Police and mental health collaborative outreach. *Psychiatric Services*, *57*(3),

417-a.

Fleischmann, M. H., Strode, P., Broussard, B., & Compton, M. T. (2018). Law enforcement officers’

perceptions of and responses to traumatic events: a survey of officers completing Crisis

Intervention Team training. *Policing and Society*, *28*(2), 149-156.

Forchuk, C., Jensen, E., Martin, M. L., Csiernik, R., & Atyeo, H. (2010). Psychiatric crisis services in

three communities. *Canadian Journal of Community Mental Health*, *29*(S5), 73-86.

Forrester, A. (2016). *Evaluating the criminal justice mental health pathway* (Master’s Thesis). King’s

College London, London, United Kingdom.

Franz, S., & Borum, R. (2011). Crisis intervention teams may prevent arrests of people with mental

illnesses. *Police practice and research: an international journal*, *12*(3), 265-272.

Godschalx, S. M. (1984). Effect of a mental health educational program upon police officers.

*Research in nursing & health*, *7*(2), 111-117.

Hanafi, S., Bahora, M., Demir, B. N., & Compton, M. T. (2008). Incorporating crisis intervention team

(CIT) knowledge and skills into the daily work of police officers: A focus group study.

*Community Mental Health Journal*, *44*(6), 427-432.

Helfgott, J. B., Hickman, M. J., & Labossiere, A. P. (2016). A descriptive evaluation of the Seattle

Police Department's crisis response team officer/mental health professional partnership pilot

program. *International journal of law and psychiatry*, *44*, 109-122.

Herrington, V., & Pope, R. (2014). The impact of police training in mental health: an example from

Australia. *Policing and society*, *24*(5), 501-522.

Hollander, Y., Lee, S. J., Tahtalian, S., Young, D., & Kulkarni, J. (2012). Challenges relating to the

interface between crisis mental health clinicians and police when engaging with people with a

mental illness. *Psychiatry, Psychology and Law*, *19*(3), 402-411.

Jennings, P., & Matheson-Monnet, C. B. (2017). Multi-agency mentoring pilot intervention for high

intensity service users of emergency public services: the Isle of Wight Integrated Recovery

Programme. *Journal of Criminological Research, Policy and Practice*, *3*(2), 105-118.

Kalinich, A. O. (2010). *Crisis Intervention Team (CIT): Perspectives from mental health professionals*.

Azusa Pacific University.

Khalsa, H. M. K., Denes, A. C., M. Pasini-Hill, D., Santelli, J. C., & Baldessarini, R. J. (2017).

Specialized Police-Based Mental Health Crisis Response: The First 10 Years of Colorado’s

Crisis Intervention Team Implementation. *Psychiatric services*, appi-ps.

King, S. M. (2011). *The impact of crisis intervention team training on law enforcement officers: An*

*evaluation of self-efficacy and attitudes toward people with mental illness* (Doctoral

dissertation, Auburn University).

Kohrt, B. A., Blasingame, E., Compton, M. T., Dakana, S. F., Dossen, B., Lang, F., ... & Cooper, J.

(2015). Adapting the Crisis Intervention Team (CIT) Model of Polic e–M ental Health

Collaboration in a Low-Income, Post-Conflict Country: Curriculum Development in Liberia,

West Africa. *American journal of public health*, *105*(3), e73-e80.

Krameddine, Y. I., & Silverstone, P. H. (2015). How to improve interactions between police and the

mentally ill. *Frontiers in psychiatry*, *5*, 186.

Krayer, A., Robinson, C. A., & Poole, R. (2018). Exploration of joint working practices on anti‐social

behaviour between criminal justice, mental health and social care agencies: A qualitative

study. *Health & social care in the community*, *26*(3), e431-441.

Kubiak, S., Comartin, E., Milanovic, E., Bybee, D., Tillander, E., Rabaut, C., ... & Schneider, S.

(2017). Countywide implementation of crisis intervention teams: multiple methods, measures

and sustained outcomes. *Behavioral sciences & the law*, *35*(5-6), 456-469.

Laing, R., Halsey, R., Donohue, D., Newman, C., & Cashin, A. (2009). Application of a model for the

development of a mental health service delivery collaboration between police and the health

service. *Issues in mental health nursing*, *30*(5), 337-341.

Lancaster, A. (2016). Evidence for joint police and mental health responses for people in mental

health crisis. *Mental Health Practice (2014+)*, *19*(10), 20.

Liegghio, M., & Jaswal, P. (2015). Police encounters in child and youth mental health: could stigma

informed crisis intervention training (CIT) for parents help?. *Journal of Social Work Practice*,

*29*(3), 301-319.

Loch, S. E. (2008). *Houston police officer attitudes towards the mentally ill: CIT trained and non-CIT*

*trained* (Doctoral dissertation, The University of Texas School of Public Health).

Lord, V. B., Bjerregaard, B., Blevins, K. R., & Whisman, H. (2011). Factors influencing the responses

of crisis intervention team–certified law enforcement officers. *Police Quarterly*, *14*(4), 388-

406.

McGuire, A. B., & Bond, G. R. (2011). Critical elements of the crisis intervention team model of jail

diversion: An expert survey. *Behavioral sciences & the Law*, *29*(1), 81-94.

Morabito, M. S., Kerr, A. N., Watson, A., Draine, J., Ottati, V., & Angell, B. (2012). Crisis intervention

teams and people with mental illness: Exploring the factors that influence the use of force.

*Crime & delinquency*, *58*(1), 57-77.

Morabito, M. S., Watson, A., & Draine, J. (2013). Police officer acceptance of new innovation: the

case of crisis intervention teams. *Policing: An International Journal of Police Strategies &*

*Management*, *36*(2), 421-436.

Mulay, A. L., Vayshenker, B., West, M. L., & Kelly, E. (2016). Crisis intervention training and implicit

stigma toward mental illness: reducing bias among criminal justice personnel. *International*

*journal of forensic mental health*, *15*(4), 369-381.

Murphy, K. (2012). Crisis Intervention teams and mobile crisis management. *Mental Health*, *73*(3),

200.

Norris, J. N. (2015). *Effects of Crisis Intervention Team Training and Simulated Auditory*

*Hallucinations on Self-efficacy, Empathy, Attitudes, and Stigma Toward Severe Mental Illness*

(Doctoral dissertation, Alliant International University, California School of Professional

Psychology, San Diego).

Oliva, J. R., & Compton, M. T. (2008). A statewide crisis intervention team (CIT) initiative: Evolution of

the Georgia CIT program. *Journal of the American Academy of Psychiatry and the Law*

*Online*, *36*(1), 38-46.

Pathé, M. T., Haworth, D. J., Goodwin, T. A., Holman, A. G., Amos, S. J., Winterbourne, P., & Day, L.

(2018). Establishing a joint agency response to the threat of lone-actor grievance-fuelled

violence. *The Journal of Forensic Psychiatry & Psychology*, *29*(1), 37-52.

Patterson, G. T. (2004). Police–social work crisis teams: Practice and research implications. *Stress,*

*Trauma, and Crisis*, *7*(2), 93-104.

Paulson, H. B. (1973). *The Role of the Community Mental [health] Nurse as Consultant to Police*

*Officers in Three California Suburban Police Departments* (Doctoral dissertation, University of

California, San Francisco).

Prince, J. L. (2013). *A phenomenological study of the impact of Crisis Intervention Team training on*

*Washington, DC police officers* (Doctoral dissertation, Argosy University/Washington DC).

Ralph, M. (2010). The impact of crisis intervention team programs: Fostering collaborative

relationships. *Journal of Emergency Nursing*, *36*(1), 60-62.

Reuland, M. M., Draper, L., & Norton, B. (2010). *Improving responses to people with mental illnesses:*

*Tailoring law enforcement initiatives to individual jurisdictions*. Justice Center, the Council of

State Governments.

RiCharde, S. G. (2016). *American and Australian Communications Officers: Mental Health Stigma*

*and Crisis Intervention Team* (Doctoral dissertation, The Chicago School of Professional

Psychology).

Richter, M. Y. (2010). *Police Response to Persons with Mental Illness by Census Tract Characters*

(Doctoral dissertation, Sam Houston State University).

Ritter, C., Teller, J. L., Marcussen, K., Munetz, M. R., & Teasdale, B. (2011). Crisis intervention team

officer dispatch, assessment, and disposition: Interactions with individuals with severe mental

illness. *International journal of law and psychiatry*, *34*(1), 30-38.

Ritter, C., Teller, J. L., Munetz, M. R., & Bonfine, N. (2010). Crisis Intervention Team (CIT) training:

selection effects and long-term changes in perceptions of mental illness and community

preparedness. *Journal of Police Crisis Negotiations*, *10*(1-2), 133-152.

Rodriguez, V. M. (2016). *The Impact of Psychiatric Emergency Response Team (PERT) Training on*

*Law Enforcement Officers on Time and Disposition Responding to Mental Health Related*

*Emergencies in Urban, Suburban, and Rural Communities* (Doctoral dissertation, Alliant

International University).

Scott, R., & Meehan, T. (2017). Inter-agency collaboration between mental health services and police

in Queensland. *Australasian Psychiatry*, *25*(4), 399-402.

Shapiro, G. K., Cusi, A., Kirst, M., O’Campo, P., Nakhost, A., & Stergiopoulos, V. (2015). Co-

responding police-mental health programs: a review. *Administration and Policy in Mental*

*Health and Mental Health Services Research*, *42*(5), 606-620.

Skeem, J., & Bibeau, L. (2008). How does violence potential relate to crisis intervention team

responses to emergencies?. *Psychiatric Services*, *59*(2), 201-204.

Skubby, D., Bonfine, N., Novisky, M., Munetz, M. R., & Ritter, C. (2013). Crisis intervention team (CIT)

programs in rural communities: a focus group study. *Community mental health journal*, *49*(6),

756-764.

Steadman, H. J., & Morrissette, D. (2016). Police responses to persons with mental illness: Going

beyond CIT training. *Psychiatric services*, *67*(10), 1054-1056.

Steadman, H. J., Deane, M. W., Borum, R., & Morrissey, J. P. (2000). Comparing outcomes of major

models of police responses to mental health emergencies. *Psychiatric Services*, *51*(5), 645-

649.

Stewart, C. (2009). *Police intervention in mental health crisis: A case study of the Bloomington Crisis*

*Intervention Team (CIT) program* (Doctoral dissertation, Indiana University).

Strauss, G., Glenn, M., Reddi, P., Afaq, I., Podolskaya, A., Rybakova, T., ... & El-Mallakh, R. S.

(2005). Psychiatric disposition of patients brought in by crisis intervention team police officers.

*Community Mental Health Journal*, *41*(2), 223-228.

Taheri, S. A. (2016). Do crisis intervention teams reduce arrests and improve officer safety? A

systematic review and meta-analysis. *Criminal Justice Policy Review*, *27*(1), 76-96.

Teller, J. L., Munetz, M. R., Gil, K. M., & Ritter, C. (2006). Crisis intervention team training for police

officers responding to mental disturbance calls. *Psychiatric services*, *57*(2), 232-237.

Tyuse, S. W. (2012). A crisis intervention team program: Four-year outcomes. *Social Work in Mental*

*Health*, *10*(6), 464-477.

Tyuse, S. W., Cooper-Sadlo, S., & Underwood, S. E. (2017). Descriptive study of older adults

encountered by crisis intervention team (CIT) law enforcement officers. *Journal of women & aging*, *29*(4), 281-293.

Watson, A. C. (2010). Research in the real world: Studying Chicago police department’s crisis

intervention team program. *Research on Social Work Practice*, *20*(5), 536-543.

Watson, A. C., Ottati, V. C., Draine, J., & Morabito, M. (2011). CIT in context: The impact of mental

health resource availability and district saturation on call dispositions. *International journal of*

*law and psychiatry*, *34*(4), 287-294.

Watson, A. C., Ottati, V. C., Morabito, M., Draine, J., Kerr, A. N., & Angell, B. (2010). Outcomes of

police contacts with persons with mental illness: The impact of CIT. *Administration and Policy*

*in Mental Health and Mental Health Services Research*, *37*(4), 302-317.

Weaver, C. M., Joseph, D., Dongon, S. N., Fairweather, A., & Ruzek, J. I. (2013). Enhancing services

response to crisis incidents involving veterans: A role for law enforcement and mental health

collaboration. *Psychological Services*, *10*(1), 66-72.

Wilson-Palmer, K., & Poole, R. (2015). Street triage for mental health crises. *British journal of nursing*,

*24*(20), 1026-1027.

Wood, J. D., & Beierschmitt, L. (2014). Beyond police crisis intervention: Moving “upstream” to

manage cases and places of behavioral health vulnerability. *International journal of law and*

*psychiatry*, *37*(5), 439-447.

Young, A. M. (2015). *Variations in Specialized Policing Response Models as a Function of*

*Community Characteristics-A Survey of Crisis Intervention Team Coordinators*. Virginia

Commonwealth University.

Young, A., Fuller, J., & Riley, B. (2008). On-scene mental health counseling provided through police

departments. *Journal of Mental Health Counseling*, *30*(4), 345-361.

Zamora, M. F. (2006). *The effect of crisis intervention team training on attitudes of Houston police*

*officers* (Doctoral dissertation, University of Houston).

Zealberg, J. J., Christie, S. D., Puckett, J. A., McAlhany, D., & Durban, M. (1992). A mobile crisis

program: collaboration between emergency psychiatric services and police. *Psychiatric*

*Services*, *43*(6), 612-615.
